# Public knowledge of emergency medicine in Beirut, Lebanon

**DOI:** 10.1186/s12873-018-0204-3

**Published:** 2018-12-13

**Authors:** Afif Mufarrij, Nicholas Batley, Rinad Bakhti, Philippe Doueihi, Hani Tamim

**Affiliations:** 1 Department of Emergency Medicine, American University of Beirut Medical Center, Beirut, Lebanon; 2 Department of Internal Medicine, Clinical Research Institute, Residency Research Program, Faculty of Medicine, American University of Beirut Medical Center, Beirut, Lebanon

**Keywords:** Emergency medicine, Public’s knowledge, Lebanon, Expectations

## Abstract

**Introduction:**

To examine the public’s level of knowledge and expectations of Emergency Medicine (EM) in Beirut, Lebanon**.**

**Methods:**

A nested cross-sectional study was conducted exploring participants’ knowledge and expectations of EM; the skillset, role and scope of practice of the emergency physician, and the dynamics of the Emergency Department (ED).

**Results:**

A majority understand EM physicians perform minor procedures (83%), have specialized training (79%) and that they should be treated by a specialized EM physician (74%). However, they also believed they should visit the ED for faster service (81%) or whenever they cannot be seen by their doctor (71%); most also expected to see their personal doctors in the ED (88%). There were significant misconceptions that ED physician could be a general doctor (84%), a specialist (81%) or a family doctor (70%). Half believe patients have the right to order blood tests (46%) or X-rays (50%) and to be admitted to the hospital at their preference (51%). Most (90%) expected patients with a possibly life-threatening problem to be treated immediately, and 48% a wait of less than thirty minutes for a non-life threatening problem. Half (54%) expected test results returned within thirty minutes, and 62% expected to spend less than sixty minutes in the ED.

**Conclusion:**

There is poor recognition of the role of the EM physician and the dynamics of the ED among the Lebanese population. Awareness campaigns targeted to improve understanding may help align expectations with the reality of the practice of EM.

## Introduction

In 1968, the American College of Emergency Physicians (ACEP) was established in the U.S., which was followed by the recognition of Emergency Medicine (EM) as a specialty status in 1979 and board certification in 1980 [1]. Across the years, the general public in the U.S. has become more aware of this specialty, though early studies conducted there have shown a relative lack of knowledge of what EM physicians actually are, what they can and cannot do and their scope of practice. A survey carried out in 1998 found that 44% of respondents thought EM physicians were not specialists, whereas only 19% thought they could achieve board certification in EM [2]. In 1999, a survey-based study looked at Emergency Department (ED) patients’ perception of the specialty of EM. It revealed that 22% believed that EM physicians have their own practice outside the ED and 26% thought that EM physicians perform surgery (eg, appendectomy and gallbladder operations). Additionally, 26% of patients expected their primary care physician to see them in the ED [3]. To the best of our knowledge, no study in Europe examined laypeople knowledge of EM as a specialty.

EM has spread as a specialty in developed and developing countries [4, 5]. Shanovich et al. assessed the public’s knowledge, attitude, and practice of emergency care in war-torn Iraq, a country with a developing emergency care system. Results indicated that most Iraqis (94%) are aware of the need for urgent care in case of serious injury but have limited knowledge of the services available.

EM is a relatively newly established specialty, especially in developing countries. It is involved in diagnosing and treating all types of emergencies which leads to an overlap between the services provided by physicians of most other specialities and those provided by the EM physician. There is a paucity of data pertaining to public knowledge about EM and the scope of practice of EM physicians in countries such as Lebanon [6]. Recent studies have shown that patient education can lead to more appropriate EM use [7]. Therefore, it is pertinent to assess the public EM knowledge in Lebanon in order to develop awareness campaigns aimed at optimizing the patient-physician relationship by aligning patients’ expectations with the services provided by EM physician. This may ultimately lead to better resource allocation and utilization. [6]. Thus, the aim of this study was to examine the public’s level of knowledge and expectations of EM in Beirut, Lebanon, as well as to assess factors associated with them.

## Methods

### Setting and design

A nested cross sectional study was conducted among a representative sample of subjects who have previously participated in a separate study with American University of Beirut - Medical Center (AUBMC). The original study was a cohort community-based study using multistage probability sampling, of a representative sample of adult subjects residing in Beirut. More specifically, a representative sample of adults in Lebanon was selected using door-to-door sampling and a multistage stratified sampling. The strata were districts of Greater Beirut, and within each district, neighborhoods, then households were selected based on a systematic random sampling. The numbers of neighborhoods and households selected within each district were proportional to the total estimated number of neighborhoods and households within this district, respectively. At the household level, the adult respondent was invited to participate, if eligible. The study was carried out at the American University of Beirut during the period ranging from February to June of 2014. Lebanese adults above age 18 years were eligible for inclusion. Vulnerable and special populations such as pregnant women, patients on dialysis, mentally disabled subjects were excluded from the study. Details of method of the original study has been published elsewhere [8–11].

The current study was carried out between May and July 2016. All participants who provided a written consent to be contacted for future research were approached. A trained research assistant carried out a phone interview with participants who agreed to take part in the current study. A standardized phone script was read and explained asking for participant’s verbal consent while emphasizing that participation is voluntary and that they have the full freedom to decline participation. Moreover, participants were assured that all data would be kept confidential and secure.

### Data collection

To develop an appropriate questionnaire in our study, we incorporated questions from published studies addressing similar topics, while also including new culture-specific questions through consensus of the members of the research group [4, 5]. The survey addressed different aspects assessing public knowledge and expectations of EM. A pilot study on 10 patients was carried out, and feedback was taken into consideration to develop the final version of the questionnaire. We did not carry out a validation of this developed questionnaire, as it was beyond the objective of the study.

The questionnaire included demographic questions such as age, gender, marital status, body mass index (BMI), income, education and crowding index. Lifestyle questions were addressed such as smoking, alcohol consumption, sleep difficulties and levels of physical activity. Comorbidity questions inquired about the presence of any cardiac disease, hypertension, diabetes, dyslipidemia, thyroid disease, cancer, history of previous cardiac catheterization, cardiac stent placement, bypass surgery, bone fracture and the presence of other diseases, specifically; stroke, arthritis, chronic bronchitis/emphysema and liver disease. Health care related questions addressed the type of health insurance, whether the insurance covers emergency care, whether the respondent has ever been to an ED (AUBMC or another hospital) and the frequency of his/her visits.

Several questions that addressed patients’ knowledge of EM were formulated. Specifically, subjects were asked whether they visited the ED because their general doctor can’t see them that day or because they are seeking a faster service. Moreover, participants’ knowledge about whether EM being a medical specialty, whether EM physicians have specialized training, and whether they have their own practice outside the ED was also sought. Questions regarding care for patients after admission and whether EM physicians perform surgery or procedures like stitching and draining abscesses were also asked.

Three categories of questions related to patients’ expectations of EM were developed. The first was “patients’ expectation of the EM physician”, which included whether respondents expect their personal doctor in the ED, whether they expect to be treated by a physician specialized in EM or a specialist related to the complaint, whether they have the right to choose the physician and whether the expected doctor in the ED is a general doctor, a specialist, an EM physician or a family doctor. The second category was related to the expectations pertaining to the services provided in the ED. It included questions related to the expectations about the number of family members allowed into the ED with the patient, whether ED patients have the right to order blood tests and x-rays at their own discretion and whether they have the right to be admitted to the hospital at their request. The third category included questions related to the time expectations in the ED, patients’ opinion about medical conditions that needs to be seen first in the ED, the expected waiting time for non-life threatening problems; the expected waiting time for possibly life threatening problems; the expected waiting time for tests and results, and the total time expected to be spent in the ED.

The answers to the above questions were specified on a 5-point Likert scale, ranging from “Strongly disagree” to “Strongly agree”. An answer was considered correct when the subject agreed with a correct statement or disagreed with an incorrect statement, whereas those who gave a neutral answer were considered wrong (refer to Table 2, as an appendix for details). Moreover, the Likert scale score was transformed to 0–100. A score of 0 indicates a wrong answer, whereas a score of 100 indicates a correct answer. This scoring system was implemented on questions inquiring about both knowledge, expectations, and an overall score including both knowledge and expectations of respondents.

### Statistical analyses

The Statistical Package for Social Sciences (SPSS), version 24, was used for the data entry, management, and analyses. Categorical variables were presented as frequencies and percentages, while continuous ones were presented as means and standard deviations.

The patients were divided into groups according to their median level of knowledge and expectations. Those above the median were considered as having a good level of knowledge or correct expectations, whereas those lower than the median were considered to have poor knowledge or false expectations. Bivariate analyses were carried out to assess the association between different demographic characteristics and knowledge and expectations and was done by chi-squared test. These demographic characteristics were: age (reference: ≤40 years); gender (reference: male); BMI (reference: < 30); crowd index (reference: < 1); marital status (reference: not married); income (reference: < 600$/month); education (reference: intermediate and below); smoking; alcohol; hypertension; diabetes; any cardiac disease; dyslipidemia; physical activity; health insurance; frequency of previous ED visits (reference: never).

To identify predictors of knowledge and expectations, multivariate logistic regression was performed considering the variables that were found to be statistically significant at the bivariate level.

Finally, Pearson correlation test was used to assess the correlation between knowledge and expectations scores. For all statistical analyses performed, values with a *P*-value ≤0.05 were considered statistically significant. Numbers were rounded to the nearest whole number for the percentages of correct and incorrect answers illustrated in Table 2.

## Results

Out of the total sample included in the original study (*n* = 501), 249 subjects accepted to participate in the study and thus included in the current analyses (response rate of 49%). Overall characteristics of study participants are presented in Table 1. A comparison with the original sample did not identify any significant differences with the sample included in this study. The mean age of the participants was 46 ± 14 years, with higher female representation (61%). The family income was < 600 US$/month for 34% of the sample, with 37% having at least an intermediate school education. Looking at respondents’ lifestyles; 47% were smokers, 29% consumed alcohol and the majority (85%) did engage in some physical activity. When asked about their medical problems, the highest prevalence was for hypertension (HTN) with 37% followed by history of having sustained a bone fracture (23%). Regarding health care coverage, a minority had private insurance (9%), whereas 42% were covered by the National Social Security Fund (NSSF) (the state-run health insurance coverage for workers).

**Table 1:** Demographics, lifestyle, comorbidities and health care coverage data of the study sample

		TOTAL *N* = 249
Demographic		
Age	Mean (±SD)	44.5 ± 13.9
	> 40	156 (62.7)
Gender	Females	152 (61.0)
Marital status	Married	173 (69.5)
BMI^a^	≥30	103 (41.4)
Income	< 600 $	77 (33.6)
	600–999.9$	81 (35.4)
	> 1000$	71 (31.0)
Education	Above intermediate	91 (36.7)
Crowding index	≥1	210 (84.3)
Lifestyle		
Smoking		116 (46.6)
Alcohol		73 (29.3)
Sleep apnea (Berlin questionnaire)	Low risk	145 (69.4)
	High risk	64 (30.6)
Sleep difficulties	Never – rarely	83 (33.3)
	Sometimes – frequently	59 (23.7)
	Almost always (16–30/ month)	107 (43.0)
Physical activity	None physical activity	38 (15.3)
	Any physical activity	211 (84.7)
Levels of physical activity	Low-intensity activity	119 (47.8)
	Moderate-intensity activity	82 (32.9)
	High-intensity activity	48 (19.3)
Insomnia		100 (40.2)
Comorbidities		
Any cardiac disease^b^		20 (8.0)
Previous cardiac catheterization		18 (7.2)
Cardiac stent placement		4 (1.6)
Cardiac Bypass surgery		1 (0.4)
Thyroid disease		26 (10.4)
Cancer		2 (0.8)
Bone fracture		58 (23.3)
Other diseases reported by a doctor		21 (8.4)
Stroke		2 (0.8)
Arthritis		45 (18.1)
Chronic bronchitis/emphysema		10 (4.0)
Liver disease		7 (2.8)
Definite diabetes		34 (13.7)
Dyslipidemia		65 (26.1)
Hypertension		92 (36.9)
Acute Disease^c^		27 (20.3)
Any disease^d^		133 (53.4)
Health care coverage		
Health insurance	No	122 (49.4)
	Private insurance	22 (8.9)
	NSSF^e^	103 (41.7)
Insurance cover emergency care		53 (54.1)
Ever been to an ED		141 (58.3)
Hospital visits	AUBMC	57 (57.0)
	Other	43 (43.0)
Frequency of hospital visits	Never	101 (45.9)
	Once	57 (25.9)
	≥ Twice	62 (28.2)

^a^BMI: Body Mass Index

^b^Any cardiac: previous cardiac catheterization, stent placed, bypass, stroke

^c^Disease acute: if any disease = yes, is it an acute one (fracture or stroke)

^d^Any disease: if any of any cardiac disease, acute disease, HTN or DM is positive

^e^NSSF: National Social Security Fund (the universal health care provided to the working class by the Lebanese government)

Table 2 presents subjects’ scores of the knowledge and expectations of EM, as well as the percent of correct answers. The highest correct knowledge score was for whether EM physicians perform procedures like stitching and draining abscesses (79 ± 25), followed by the knowledge of whether EM physicians have special training (77 ± 29). The lowest correct score was for whether patients should visit ED for faster service (27 ± 29) and whether they should seek ED care whenever they cannot be seen by their doctor (35 ± 33). When asked about their expectations related to EM physicians, 74% had a correct expectation of being examined and treated by a physician specialized in EM. However, the majority (88%) of respondents expected to see their personal doctors in the ED and had false expectations regarding the specialty of the ED physician; that the ED physician could be a general doctor (84%), a specialist (81%) or a family doctor (70%).

**Table 2:** Subjects’ scores of the responses towards the knowledge and expectations of the services provided in the ED, as well as the percent of correct answers

	Total *N* = 249	Answer		
		Expected	Right N (%)	Wrong N (%)
Total overall score	52.7 ± 9.3			
Total knowledge score	55.0 ± 12.0			
I should visit ED if my general doctor can’t see me that day	35.1 ± 33.1	No	72 (29)	177 (71)
I should visit ED for faster service	27.1 ± 29.1	No	47 (19)	202 (81)
ED is a medical specialty	59.0 ± 36.4	Yes	127 (51)	122 (49)
ED doctor have special training	76.8 ± 29.0	Yes	195 (78)	54 (22)
ED doctor care for patients after admission	54.5 ± 32.2	No	135 (54)	114 (46)
ED doctor have their own practice outside ED	49.5 ± 30.0	No	94 (38)	155 (62)
ED doctor perform surgery	58.4 ± 31.7	No	134 (54)	115 (46)
ED doctor perform procedures like stitching and draining abscesses	79.2 ± 24.8	Yes	206 (83)	43 (17)
Total expectation score	51.6 ± 11.3			
Total expectation physician score	45.3 ± 11.3			
Personal doctor expected in ED	33.5 ± 30.4	No	55 (22)	194 (78)
In ED I should be treated by an ED doctor specialized in EM	72.3 ± 30.4	Yes	181 (73)	68 (27)
In ED I should be treated by a specialist related to the complaint	22.8 ± 29.7	No	34 (14)	215 (86)
I expect ED physician to be a General doctor	27.5 ± 28.0	No	38 (15)	211 (85)
I expect ED physician to be a Specialist	36.2 ± 30.8	No	70 (28)	179 (72)
I expect ED physician to be an EM physician	85.0 ± 20.8	Yes	214 (86)	35 (14)
I expect ED physician to be a Family doctor	49.5 ± 30.9	No	103 (41)	146 (59)
I expect to see a physician of choice at my request	35.8 ± 33.6	No	72 (29)	177 (71)
Total expectation service score	59.3 ± 26.7			
Family members allowed in ED	80.7 ± 39.5	1 or 2	196 (79)	53 (21)
I have the right to order blood test at my request	53.0 ± 39.2	No	130 (52)	119 (48)
I have the right to order X rays at my request	51.9 ± 38.5	No	121 (49)	128 (51)
I have the right to get admitted to hospital at my request	51.4 ± 38.5	No	119 (48)	130 (52)
Total expectation time score	55.6 ± 23.2			
Which condition should be seen first in ED	57.0 ± 49.6	Most serious	137 (55)	112 (45)
Possibly life threatening problem	90.4 ± 29.6	Immediately	216 (87)	33 (13)
Non-life threatening problem	48.2 ± 50.1	< 30 min	115 (46)	134 (54)
Tests and results	45.4 ± 49.9	< 30 min	98 (39)	151 (61)
Total time expected to spend in ED	36.9 ± 48.4	< 60 min	72 (29)	177 (71)

Concerning patients’ expectations of the services provided in the ED, most respondents (81%) expected up to 2 family members to be allowed in the ED with a given patient admission to the ED; a correct answer as considered by the authors (Table 2). However, around half (46%) of the respondents thought that ED patients have the right to order blood tests or X-rays (50%) and to request their admission to the hospital if they want to, regardless of the physician’s recommendation (51%). Respondent’s time expectations were also addressed. Results showed that most participants (90%) correctly expected patients with a possibly life-threatening problem to be taken immediately to treatment room, and 48% correctly expected a waiting time of less than 30 min for patients with a non-life threatening problem. On the other hand, 54% of subjects expected tests to be complete within 30 min, and 62% expected to spend no more than 60 min in total in the ED; both answers considered incorrect by the authors.

The association between the overall knowledge and expectation score, as categorized by the median, and the different baseline demographic, lifestyle, comorbidities and health care coverage are presented in Table 3. It was found that education above intermediate school level was the only variable associated with the total overall score (*p* = 0.005). Although not statistically significant, it was found that younger people, males, and non-obese subjects with NSSF coverage were more likely to have a higher overall score.

**Table 3:** The association between the overall knowledge and expectation score (categorized according to the median) with baseline demographic, lifestyle, comorbidities, and health care coverage characteristics

		Total overall score		
		< 53.00 *N* = 127	≥53.00 *N* = 122	*p*-value
Age	> 40	84 (66.1)	72 (59.0)	0.24
Gender	Male	46 (36.2)	51 (41.8)	0.37
Marital status	Married	90 (70.9)	83 (68.0)	0.63
BMI	≥30	58 (45.7)	45 (36.9)	0.16
Income	< 600$	42 (36.2)	35 (31.0)	0.15
	600 - < 1000$	34 (29.3)	47 (41.6)	
	≥1000$	40 (34.5)	31 (27.4)	
Education	Above intermediate	36 (28.3)	55 (45.5)	0.005
Crowd index	≥1	111 (87.4)	99 (81.1)	0.17
Smoking	Ever	58 (45.7)	58 (47.5)	0.76
Alcohol	Ever	33 (26.0)	40 (32.8)	0.24
Any cardiac disease		12 (9.4)	8 (6.6)	0.40
Disease Acute		14 (11.0)	13 (10.7)	0.93
Any disease		75 (59.1)	58 (47.5)	0.07
Dyslipidemia		38 (29.9)	27 (22.1)	0.16
Hypertension		53 (41.7)	39 (32.0)	0.11
Definite diabetes		20 (15.7)	14 (11.5)	0.33
Sleep apnea (Berlin questionnaire)	Low risk	75 (67.6)	70 (71.4)	0.55
	High risk	36 (32.4)	28 (28.6)	
Sleep difficulties	Never – rarely	34 (26.8)	49 (40.2)	0.06
	Sometime – frequently	31 (24.4)	28 (23.0)	
	Almost always	62 (48.8)	45 (36.9)	
Physical activity	None	21 (16.5)	17 (13.9)	0.57
	Any physical activity	106 (83.5)	105 (86.1)	
Levels of physical activity	Low-intensity activity	62 (48.8)	57 (46.7)	0.52
	Moderate-intensity activity	38 (29.9)	44 (36.1)	
	High-intensity activity	27 (21.3)	21 (17.2)	
Insomnia		55 (43.3)	45 (36.9)	0.30
Health insurance	No	70 (55.6)	52 (43.0)	0.14
	Private insurance	10 (7.9)	12 (9.9)	
	NSSF	46 (36.5)	57 (47.1)	
Insurance cover emergency care		25 (59.5)	28 (50.0)	0.35
Ever been to an ED		77 (61.1)	64 (55.2)	0.35
Hospital visits	AUBMC	36 (58.1)	21 (55.3)	0.78
	Other	26 (41.9)	17 (44.7)	
Frequency of visits	Never	49 (41.9)	52 (50.5)	0.20
	Once	36 (30.8)	21 (20.4)	
	≥ Twice	32 (27.4)	30 (29.1)	

The results of the multivariate analyses for the predictors of the different knowledge and expectation scores are presented in Table 4. It was found that education above intermediate school level was found to significantly predict a higher total overall score [OR (95% CI): 2.14 (1.26–3.64), p = 0.005]. On the other hand, subjects’ income (≥$1000/month) was found to be significantly associated with lower total expectation score (p = 0.005), whereas education above intermediate school level was found to be associated with higher total expectation score (*p* = 0.004). As for the total expectation physician score, education above intermediate school level was found to be the only significant predictor of a higher score (*p* = 0.02). Both education above intermediate school level and NSSF health coverage were found to be significant predictors of higher total expectation service score, whereas subjects’ income (≥$1000$/month) was associated with lower score. Finally, none of the baseline characteristics was found to significantly predict participants’ total expectation time score or total knowledge score.

**Table 4:** Multivariate analysis of potential predictors of the different knowledge and expectation scores

Variables	OR (95% CI)	*P*-value
Total overall score (reference: < 53.00)		
Education – above intermediate	2.14 (1.26–3.64)	0.005
Total expectation score (reference: ≤52.93)		
Income – ≥1000$	0.40 (0.22–0.76)	0.005
Education – above intermediate	2.39 (1.32–4.33)	0.004
Total expectation physician score (reference: ≤43.74)		
Education – above intermediate	1.85 (1.08–3.17)	0.02
Total expectation service score (reference: < 62.50)		
Income – ≥1000$	0.51 (0.28–0.93)	0.03
Education – above intermediate	1.93 (1.09–3.42)	0.02
Health insurance - NSSF	1.84 (1.09–3.10)	0.02
Total expectation time score (reference: < 60.00) None		
Total knowledge score (reference: < 56.25) None		

Variables included in the model were:

Age (reference: ≤40); gender (reference: male); BMI (reference: < 30); Crowding index (reference: < 1); marital status (reference: non-married); income (reference: < 600$); education (reference: intermediate and below);smoking (reference: never); drinker alcohol (reference: never); HTN (reference: no); Diabetes (reference: no); any cardiac disease (reference: no); Dyslipidemia (reference: no); Physical activity (reference: none Physical activity); health insurance (reference: No); how many times ever been to an ED (reference: never)

## Discussion

This study was a cross-sectional assessment of the Lebanese public’s knowledge and expectations’ level of EM in Lebanon. The survey was carried out on a representative sample of residents in Beirut who were above 18 years of age. Our results revealed a relatively poor knowledge of EM, as well as poor expectations of the functioning of the EM system in general.

This study revealed that a high number of respondents failed to recognize the exact practice and tasks of EM trained physicians. We found that around half (49%) of study subjects believed that EM is not a medical specialty. This is consistent with the results reported by two studies carried out in the United States around 2 decades ago: Cordell et al. (1998) reported that 44% of laypeople think that ED physicians are not specialists [2], while Olsen et al. (2000) reported 38% perceived EM not to be a medical specialty [3]. As for another misconception about the role of EM physicians, 55% of our study subjects wrongly believed that EM physicians care for patients on the hospital inpatient ward after admission, as compared to only 19% in the same study conducted by Olsen et al. [3]. Although, to the best of our knowledge, we were not able to find a recent study carried out in the U.S. reporting on what general knowledge about EM is, one would expect that a more recent study should yield better knowledge about the specialty. Our results show that a large number of people in Lebanon look at EM as an ill-defined specialty and have an inaccurate assessment of the scope of practice of EM physicians. These results are consistent with the findings in the U.S. 20 years ago, which could be attributed to the fact that EM in Lebanon is still in its formative years [6]. Our findings in Lebanon could be attributed to the fact that EM specialty has been recently introduced to the Lebanese medical system, where historically emergency units in Lebanon have been run by non-EM specialists or general physicians.

The vast majority of our participants failed to correctly answer questions about ED functioning. Around 80% of respondents believed they should visit the ED for faster service which is significantly higher than what is reported in the literature. A study conducted in Washington found that the leading factor (24%) driving non-urgent ED use was “ease of use” [12]. A study conducted for California Healthcare Foundation (2006) found that 50% of ED users cited easier access to diagnostic testing as a major factor in decision to go to ED [13]. On the other hand, we found that 71% of participants reported going to the ED if they could not be seen by their general doctor on a specific day. This may reflect limitations Lebanese people face in accessing primary care in an ill-defined primary health care system in Lebanon, as well as poor health insurance coverage. The poorly-structured primary health care system in Lebanon could be due to the civil war and its repercussions on state-run institutions. The perception that these services can be accessed faster through the ED is a typical example of a major inappropriate use of ED resources. Comparable conclusions could be drawn from a study conducted in the US (2002) showing that difficulty getting an appointment, long waiting times and difficulty talking with the usual source of care over the phone, were all significantly associated with non-urgent ED visits [14].

When asked about the expectation of the EM physicians’ specialty, our study revealed that a high number of subjects had false expectations that an EM physician could be a general doctor (84%), a specialist (71%) or a family doctor (57%). A majority (78%) also expected their personal doctor to be present for a consultation in the ED. In comparison, Olsen et al. (United States, 2000) found that 26% of patients with primary care physicians expected to be seen by their primary care physician in the ED [3], whereas Cooke et al. (Canada, 2002) reported that 12% confirmed attending the ED to see a non-EM specialist [15]. Our results highlight the Lebanese public’s inappropriate understanding of the EM physicians’ specialty. However, it is noteworthy to mention that in Lebanon most of the EDs are run by physicians who do did not go through EM specialty training, which could explain why the majority of our study subjects are inclined to have these expectations.

The survey also addressed patients’ expectations regarding the services provided in the ED. We found that around half of the study subjects expected ED physicians to order blood tests or imaging studies at the patient’s request, regardless of the physician’s recommendations. Similar results, albeit with some variance, were reported by Cooke et al. who found that nearly 40% of study subjects in the U.S. expected ED physicians to order tests at the patient’s request [15].

Subjects’ expectations about ED prioritization of patients based on their perception of acuity were also assessed. We found that more than half of respondents (57%) expected patients with the most serious medical condition to be seen first as compared to 69% reported in a study conducted by Cooke et al. in the U.S. [15]. We found that 90% of subjects believed patients with a life threatening problem should be attended to immediately which compares with the U.S. population where 91% believed such a patient should be seen in less than 15 min, and 61% in less than 5 min [15]. Unfortunately, there are few studies that report ED waiting time expectations by patients for comparison purposes. In addition, to the best of our knowledge, the only established guideline for ED waiting times are those set by the Canadian Emergency Department Triage and Acuity Scale (CTAS). According to these guidelines, the ideal time from triage to physician initial assessment should be 30 min or less for urgent patients (CTAS III), which corresponds to “possibly life threatening” in our study [16].

We found that an education level above intermediate/middle school was the only significant predictor of better EM knowledge and expectation scores. We found no other studies addressing predictors of EM knowledge and expectations.

### Limitations

This investigation is believed to be the only study in the region that has attempted to assess the public’s level of knowledge and expectations of EM; the skillset, role and scope of practice of the EM physician, as well as the dynamics and characteristics of the ED and the services it offers. A strength of our study was the fact that we did not include actual ED patients since patients interviewed in the ED might be biased by their medical condition, emotional status, and the people accompanying them. One limitation of our study is that we used a questionnaire that was not validated but was developed based on multiple published surveys that addressed similar questions, as well as consensus among the research group to identify setting-specific factors. Another potential weakness is the time gap between the formation of the original cohort of subjects and our study; that some of their characteristics may have changed. Finally, this study includes a relatively small sample size in one specific geographic region, which affects the representativeness of the sample, since subjects were all from the greater Beirut area.

## Conclusions

In summary, there is still poor recognition of the skillset, role and scope of practice of the EM physician, as well as the dynamics and characteristics of the ED and the services it offers among the Lebanese population. Accordingly, awareness campaigns could be implemented at multiple levels, to target the general population. Such campaigns could be developed through schools, universities, institutions, through medical care facilities, as well as governmental and non-governmental organizations. Finally, media has an integral role in disseminating information and raising public knowledge of EM speciality and services (an Arab “ER” TV show?). In view of the current overcrowding of EDs in Lebanon, increasing knowledge and awareness among the general public would improve the allocation of limited emergency health resources. Further research is needed to assess the knowledge and expectations of EM among the wider Lebanese population and throughout the region.

## Abbreviations

ACEP: American College of Emergency Physicians
BMI: Body mass index
CTAS: Canadian Emergency Department Triage and Acuity Scale
ED: Emergency Department
EM: Emergency Medicine
HTN: Hypertension
NSSF: National Social Security Fund
